# MGCF-Net: a multi-view granular-ball contrastive fusion network for spatial transcriptomics clustering

**DOI:** 10.3389/fphys.2026.1967219

**Published:** 2026-10-07

**Authors:** Li Zhu, Haiyao Dong, Yuji Zhang, Yong Zhang, Xinyi Huang, Huishan Wang

**Affiliations:** 1 Graduate School of Dalian Medical University, Dalian, Liaoning, China; 2 State Key Laboratory of Frigid Zone Cardiovascular Disease, Department of Cardiovascular Surgery, General Hospital of Northern Theater Command, Shenyang, Liaoning, China; 3 Postgraduate College, China Medical University, Shenyang, Liaoning, China

**Keywords:** granular-ball contrastive learning, graph convolutional networks, multi-view graph learning, spatial domain identification, spatial transcriptomics

## Abstract

**Introduction:**

Spatial domain identification from spatial transcriptomics (ST) data is essential for characterizing the spatial organization of complex tissues. Existing contrastive learning methods face a persistent trade-off: instance-level approaches are susceptible to false negatives, whereas cluster-level methods may overlook fine-grained local structures. Moreover, direct multi-view fusion may introduce redundant or noisy correlations, thereby degrading representation quality.

**Methods:**

To address these limitations, we propose MGCF-Net, a multi-view granular-ball contrastive fusion network for spatial domain identification. MGCF-Net constructs a spatial graph and a feature graph to represent physical proximity and transcriptomic similarity, respectively. A redundancy-reduction objective preserves spot-level correspondence across the two views while suppressing spurious similarities between different spots. Multi-view granular-ball contrastive learning groups spot-level representations into local structural units and aligns them across views according to their membership overlap.

**Results:**

On eight dorsolateral prefrontal cortex (DLPFC) tissue sections, MGCF-Net attains the highest mean adjusted Rand index (ARI) among the evaluated methods. Its normalized mutual information (NMI) remains competitive with the comparison methods.

**Discussion:**

By contrasting local structural units rather than individual spots, MGCF-Net reduces the influence of false negatives arising from instance-level contrastive learning. This group-level formulation preserves coherent spatial structures without requiring a generative decoder.

## 1 Introduction

Spatial transcriptomics (ST) enables the joint characterization of gene expression and spatial organization within complex tissues Ståhl et al. (2016); Rodriques et al. (2019); Vickovic et al. (2019); Stickels et al. (2021). A fundamental task in ST analysis is spatial domain identification, which partitions tissue sections into regions with coherent transcriptional profiles and spatial structures. Accurate domain identification is essential for characterizing tissue architecture, revealing cellular interactions, and investigating diseaseassociated microenvironments Longo et al. (2021); Moses and Pachter (2022).

Discriminative representation learning is also central to biomedical and biometric image analysis. The shift from handcrafted descriptors to learned features is documented in neural-network-based finger vein recognition (Yin et al., 2025). Few-shot class-incremental learning addresses a different constraint: incorporating new classes from limited labeled examples while retaining earlier knowledge (Zhang et al., 2025b). For pill recognition, WP-FSCIL and DBC-FSCIL combine discriminative feature learning with mechanisms for preserving knowledge of previously learned classes (Zhang et al., 2026, Zhang et al., 2025a). Re-FSCIL combines supervised contrastive learning and feature fusion for retinal disease recognition (Zhang et al., 2025c). These label-driven recognition settings differ from spatial domain identification, which seeks tissue partitions that reflect molecular similarity and spatial organization.

Recent ST clustering methods increasingly employ graph neural networks (GNNs) to model spatial proximity and transcriptional similarity Hu et al. (2021); Dong and Zhang (2022); Long et al. (2023). These methods typically construct a spatial graph from physical coordinates and a feature graph from gene expression profiles, followed by graph-based representation learning and multi-view fusion. Many existing frameworks further adopt generative decoders, such as zero-inflated negative binomial models, to reconstruct sparse expression counts Lopez et al. (2018); Eraslan et al. (2019). Alternatively, contrastive learning has been introduced to align representations across graph views without explicitly modeling the count distribution Chen et al. (2020); Long et al. (2023).

Despite their effectiveness, existing approaches exhibit two major limitations. First, direct multi-view fusion often emphasizes cross-view consensus while insufficiently preserving view-specific structures. Repeated information propagation across graph views may homogenize latent representations and obscure discriminative patterns that are unique to either the spatial or transcriptomic view. The fused embedding may therefore retain redundant correlations while discarding view-specific structure Zbontar et al. (2021); Bardes et al. (2022).

Second, current optimization strategies predominantly operate at the individual-spot level. Count reconstruction encourages spot-level expression fidelity but provides limited supervision for spatial domain boundaries. Similarly, instance-level contrastive learning treats individual spots as independent contrastive units and commonly assumes that unmatched samples constitute negative pairs. This assumption is unreliable in heterogeneous tissues, where nearby spots from the same biological domain may be incorrectly separated as false negatives Chuang et al. (2020); Huynh et al. (2022). Such spot-level objectives may therefore disrupt local spatial continuity and fail to capture higher-level tissue organization.

To address these limitations, we propose the Multi-View Granular-Ball Contrastive Fusion Network (MGCF-Net), a decoder-free framework for spatial domain identification. MGCF-Net constructs a spatial graph and a feature graph to characterize physical proximity and transcriptomic similarity. Two independent GCN encoders preserve the topology of each view, while an additional encoder operates on a learnably fused graph to capture their shared structure. A multi-view redundancy reduction (MVRR) objective aligns representations of corresponding spots while suppressing spurious similarities between different spots. The resulting spatial, transcriptomic, and joint representations are subsequently integrated through a spot-adaptive gating mechanism.

The central component of MGCF-Net is a multi-view granular-ball contrastive learning (GBCL) module. Rather than contrasting individual spots, GBCL groups locally similar spots into granular balls and performs representation alignment between structural units. Granular balls are independently constructed in each view and associated according to their membership overlap, thereby avoiding the unreliable assumption that all unmatched spots are negatives. This group-level formulation provides more stable contrastive supervision, reduces the influence of false-negative pairs, and encourages the preservation of mesoscopic tissue structures. In addition, physical consistency refinement (PhCR) preserves spatial neighborhood relationships in the fused embedding.

The main contributions of this work are summarized as follows:

- We propose MGCF-Net, a decoder-free multi-view graph learning framework that jointly models spatial proximity, transcriptomic similarity, and their shared structural information for spatial domain identification.
- We develop a redundancy-aware fusion strategy that combines independent view-specific encoders with a learnably fused graph encoder. The proposed cross-view objective preserves spot correspondence while suppressing spurious inter-spot correlations, and the adaptive gating mechanism integrates the resulting representations at the spot level.
- We introduce a multi-view granular-ball contrastive learning module that performs cross-view alignment over local structural units rather than individual spots. By associating granular balls through membership overlap, the proposed formulation alleviates false-negative pairings and improves the spatial coherence and boundary discrimination of the learned representation. Experiments on eight DLPFC tissue sections assess the complete model, its components, parameter sensitivity, and computational cost.

## 2 Related work

### 2.1 Graph-based spatial domain identification

Encoder design provides a useful perspective on single-cell clustering. A recent survey organizes methods into embedded, topological, and multi-modal encoder families, distinguishing how they capture feature structure, cell relationships, and cross-modality information (Hu et al., 2026). DGAN-MPCC combines adversarial learning at the input and bottleneck levels with a contrastive objective that assigns multiple positives from the inferred clusters (Wang et al., 2026). These approaches focus on molecular representations, whereas spatial domain identification must also account for the physical organization of tissue.

Spatial domain identification aims to partition a tissue section into regions with coherent molecular profiles and spatial organization. Early spatially aware methods incorporated local context through probabilistic priors. BayesSpace encourages neighboring spots to share domain labels within a Bayesian framework, whereas BASS extends probabilistic modeling to multi-scale and multi-sample analysis Zhao et al. (2021); Li and Zhou (2022). SpaGCN constructs a graph that jointly represents gene expression, spatial coordinates, and histological information Hu et al. (2021). These methods established spatial context as an integral component of clustering rather than a *post hoc* correction.

Deep graph models subsequently shifted the focus toward representation learning. STAGATE learns adaptive weights between neighboring spots through a graph attention autoencoder, while SpaceFlow combines spatial graphs with self-supervised learning and spatial regularization Dong and Zhang (2022); Ren et al. (2022). DeepST learns spatially informed embeddings through deep representation learning, whereas GraphST uses graph self-supervision for clustering, integration, and deconvolution Xu et al. (2022); Long et al. (2023). SCGDL applies graph deep learning to spatial domain identification Liu et al. (2023). Several of these models preserve spot-level information through graph autoencoding or feature reconstruction, while contrastive approaches learn structural consistency without explicitly modeling the count distribution.

Recent studies have expanded the number of views and information sources used to characterize tissue structure. stLearn incorporates tissue morphology, while Spatial-MGCN constructs separate graphs to represent spatial adjacency and transcriptomic similarity Pham et al. (2023); Wang et al. (2023). stMMR integrates multimodal features into a shared representation, and BANKSY combines each spot with information from its spatial neighborhood Zhang et al. (2024); Singhal et al. (2024). PROST connects spatially variable gene detection with domain segmentation, whereas IRIS incorporates single-cell references to identify biologically interpretable domains Liang et al. (2024); Ma and Zhou (2024). STAIG further combines histological features with graph contrastive learning for domain exploration and multislice integration Yang et al. (2025). Together, these methods extend spatial domain analysis beyond a single graph by coordinating physical, molecular, and histological information.

Despite these advances, graph construction and multi-view integration remain challenging. Neighborhood graphs based on fixed radii or fixed numbers of neighbors may connect spots across tissue boundaries and fail to accommodate variations in local density. Direct graph fusion may also introduce redundant or conflicting correlations that obscure view-specific information. Moreover, spot-level contrastive objectives can incorrectly treat biologically related spots as negative pairs. Effective spatial representations therefore need to preserve view-specific graph structures while providing stable supervision for local tissue organization.

### 2.2 Multi-view granular-ball clustering

Multi-view clustering seeks a common partition from heterogeneous representations of the same samples. An effective model must exploit shared information without suppressing structures that remain specific to individual views. Instance-level objectives preserve fine details but may construct false-negative pairs, whereas cluster-level objectives provide stronger abstraction at the cost of meaningful intra-cluster variation. This trade-off motivates structural units whose granularity lies between individual samples and complete clusters.

Granular-ball computing provides such an intermediate structural unit. It organizes locally similar samples into adaptive granular balls and describes each ball through compact information such as its center, radius, and membership. This coarse-to-fine representation reduces the number of computational units and limits the influence of local noise and perturbations Xia et al. (2019). Granular balls can therefore preserve local structure without relying entirely on sample-level optimization.

Granular-ball representations have subsequently been incorporated into several clustering paradigms. GBSC constructs a compact similarity matrix from granular balls to improve the scalability of spectral clustering Xie et al. (2023). GB-DP performs density-peak clustering at the granular-ball level, whereas GB-DBSCAN defines density connectivity between granular balls Cheng et al. (2024a; Cheng et al. (2024b). MGNR further introduces multi-granularity neighbor relationships for classification and clustering Xie et al. (2024). These methods retain local clustering structure while reducing the computational cost and noise sensitivity of sample-level analysis.

Recent studies have extended granular-ball learning to multi-view settings. MGBCC constructs granular balls independently in each view and establishes cross-view associations according to membership overlap Su et al. (2025). GB-MKKM introduces granular-ball representations into multiple-kernel clustering to improve computational efficiency and robustness Xia et al. (2025). GFSAF uses fuzzy granular-ball contrastive learning to separate shared and complementary representations before attention-based view fusion Liu et al. (2026). HOARD combines granular-ball contrastive alignment with hierarchical cross-view information transfer Zhou et al. (2026). These developments position granular balls as trainable local structural units rather than simple data-compression devices.

Existing multi-view granular-ball methods have mainly been evaluated on generic benchmark data, where different views usually correspond to alternative feature descriptions. In spatial transcriptomics, the spatial graph and feature graph instead encode distinct physical and molecular dependencies. Tissue boundaries, physical continuity, and variations in local density can affect both granular-ball construction and cross-view association. Coordinating these heterogeneous graph structures at the granular-ball level therefore remains insufficiently studied.

## 3 Methods

MGCF-Net learns from a spatial graph $G_{1}$ and a feature graph $G_{2}$, which encode physical proximity and transcriptomic similarity, respectively. The fused graph $G_{3}$ combines their adjacency matrices using learnable weights. An inter-view graph convolutional network (GCN) operates on $G_{3}$, whereas two intraview GCN encoders preserve the topology of each input graph Kipf and Welling (2017). MVRR suppresses redundant cross-view correlations before spot-adaptive fusion. The GBCL module then organizes locally similar spots into granular balls and applies contrastive learning at the group level. This design limits falsenegative pairings in instance-level contrastive learning and removes the need for a spot-level generative decoder. PhCR further preserves spatial neighborhood relationships in the fused representation. The overall architecture of MGCF-Net is illustrated in **Figure 1**.

**Figure 1:**
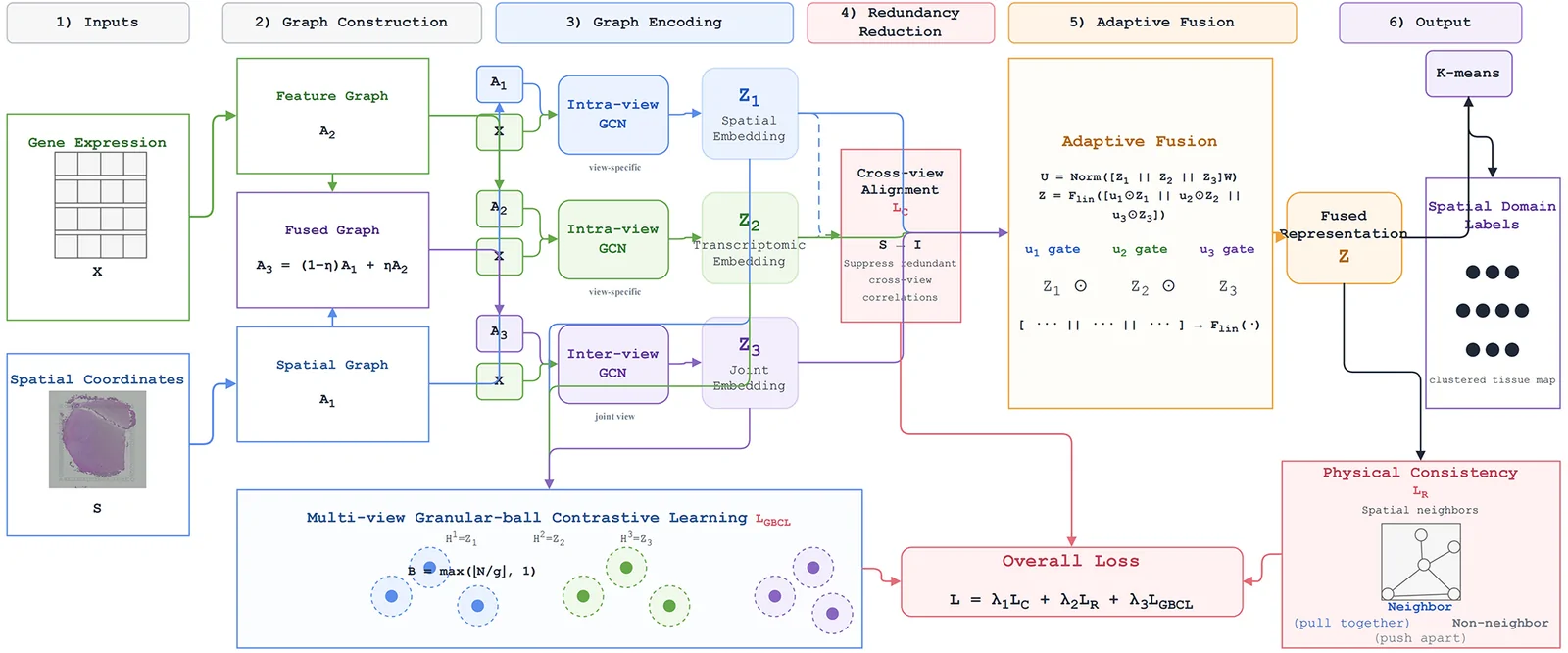
Overview of the proposed MGCF-Net framework. MGCF-Net constructs a spatial graph and a feature graph from spatial transcriptomics data and processes them with multiple graph encoders. MVRR suppresses redundant cross-view correlations, while adaptive fusion integrates view-specific and joint representations. GBCL captures local structural consistency across views, and PhCR preserves spatial neighborhood relationships. The final representation is clustered to identify spatial domains.

### 3.1 Problem formulation and preliminaries

Spatial transcriptomics data provide the spatial coordinates and gene expression profile of each spot. We represent these two sources as a spatial graph and a feature graph to characterize their respective structural dependencies. For the spatial graph $G_{1}=(A_{1},X)$, the adjacency matrix $A_{1}\in {\mathbb{R}}^{N\times N}$ is constructed from the pairwise Euclidean distances between the *N* spots. Specifically, ${\left(A_{1}\right)}_{ij}=1$ if the Euclidean distance between spots *i* and *j* is smaller than a predefined radius *r*, and ${\left(A_{1}\right)}_{ij}=0$ otherwise. The normalized gene expression matrix is denoted by $X\in {\mathbb{R}}^{N\times D}$, where *D* is the number of selected genes.

The feature graph $G_{2}=(A_{2},X)$ is constructed using the *k*-nearest-neighbor (KNN) method in the gene expression space. To construct $A_{2}\in {\mathbb{R}}^{N\times N}$, we compute the pairwise cosine similarity $\text{cos }(x_{i},x_{j})$ between the expression profiles $x_{i}$ and $x_{j}$. Accordingly, ${\left(A_{2}\right)}_{ij}=1$ if spot *j* is among the *k* most similar neighbors of spot *i*, and ${\left(A_{2}\right)}_{ij}=0$ otherwise.

### 3.2 Multi-view redundancy reduction and feature fusion

To combine the spatial and transcriptomic views, we construct a fused graph $G_{3}=(A_{3},X)$ through a learnable weighted combination of $G_{1}$ and $G_{2}$. The adjacency matrix of $G_{3}$ is defined in Equation (1) as follows

(1) $$A_{3}=(1-\eta )A_{1}+\eta A_{2},$$

where *η* is a learnable mixing coefficient that adaptively weights spatial proximity and transcriptomic similarity. It is initialized to 0.1 and optimized jointly with the other model parameters, with clipping to [0,1] applied only at initialization. A GCN encoder is then applied to $G_{3}$, yielding a joint representation *Z*_3_ that integrates the structural dependencies of both views.

Direct graph fusion may obscure view-specific information. The MVRR module therefore applies two independent GCN encoders to $G_{1}$ and $G_{2}$ to obtain the spatial representation *Z*_1_ and the transcriptomic representation *Z*_2_, respectively. To preserve spot-level correspondence while suppressing redundant cross-view correlations, MVRR regularizes the cross-view similarity matrix toward the identity matrix. Specifically, the (*i,j*)-th entry of the similarity matrix is defined in Equation (2) as follows

(2) $$S_{ij}=\frac{z_{1,i}^{⊤}z_{2,j}}{\|z_{1,i}{\|}_{2}\|z_{2,j}{\|}_{2}},$$

where *z*_1_*_,i_* and *z*_2_*_,j_* are the embeddings of spots *i* and *j* in *Z*_1_ and *Z*_2_, respectively, and $S\;\in \;{\mathbb{R}}^{N\times N}$ is the resulting cross-view similarity matrix. The MVRR loss is defined in Equation (3) as follows

(3) $${ℒ}_{\text{C}}=\frac{1}{N}\sum_{i=1}^{N}{\left(S_{ii}-1\right)}^{2}+\frac{1}{N\left(N-1\right)}\sum_{i=1}^{N}\sum_{\begin{array}{l} j=1\\ j\neq i \end{array}}^{N}S_{ij}^{2}$$

The diagonal term aligns the representations of corresponding spots across views, whereas the off-diagonal term suppresses redundant cross-view correlations between different spots.

Finally, we adaptively fuse the spatial, transcriptomic, and joint representations. Let *d* denote the output dimension shared by the three encoders, so $Z_{1},Z_{2},Z_{3}\in {\mathbb{R}}^{N\times d}$. The three representations are first concatenated as shown in Equation (4)

(4) $$Z^{\prime }=[Z_{1}\|Z_{2}\|Z_{3}]\in {\mathbb{R}}^{N\times 3d}.$$

The concatenated representation is then projected to obtain spot-specific gating coefficients are obtained as shown in Equation (5):

(5) $$U={\text{Norm}}_{l2}(Z^{\prime }W)=[u_{1}\|u_{2}\|u_{3}],$$

where $W\in {\mathbb{R}}^{3d\times 3}$ is a learnable projection matrix and $U\in {\mathbb{R}}^{N\times 3}$ contains the spot-specific gating coefficients. The operator ${\text{Norm}}_{l2}(\cdot )$ denotes row-wise $l_{2}$ normalization, and each column $u_{m}\in {\mathbb{R}}^{N\times 1}$ contains the coefficients for the *m*-th representation. Using these coefficients, the final representation is computed as shown in Equation (6)

(6) $$Z=F_{\text{lin}}\;([u_{1}⊙Z_{1}\|u_{2}⊙Z_{2}\|u_{3}⊙Z_{3}]),$$

where each coefficient vector is broadcast along the feature dimension and ⊙ denotes element-wise multiplication. The concatenated weighted features form an input matrix of size N×3d. The linear layer $F_{\text{lin}}:{\mathbb{R}}^{3d}\to {\mathbb{R}}^{d}$ acts on each spot independently, yielding the final representation $Z\in {\mathbb{R}}^{N\times d}$.

### 3.3 Multi-view granular-ball contrastive learning

Although count-based reconstruction preserves spot-level expression fidelity, it provides limited supervision for maintaining structural consistency across graph views. We therefore use GBCL to perform contrastive optimization over local structural units rather than individual spots. The three view representations are defined in Equation (7):

(7) $$H^{1}=Z_{1},\;H^{2}=Z_{2},\;H^{3}=Z_{3}.$$

These representations form the multi-view set $H={\left\{H^{v}\right\}}_{v=1}^{V}$, where *V* = 3.

Granular-Ball Construction. Each view is independently clustered using *k*-means Lloyd (1982). Let *g* denote the granularity parameter controlling the approximate number of spots per ball. The number of granular balls is defined in Equation (8)

(8) $$B=\text{max }\left(\left\lfloor \frac{N}{ɡ}\right\rfloor ,1\right).$$

Let $y_{q}^{v}$ denote the granular-ball assignment of spot *q* in view *v*. The membership set, cardinality, and center of each granular ball are defined in Equation (9)

(9) $$I_{i}^{v}=\{q|y_{q}^{v}=i\},\;n_{i}^{v}=|I_{i}^{v}|,\;c_{i}^{v}=\frac{1}{n_{i}^{v}}\sum_{q\in I_{i}^{v}}h_{q}^{v},$$

where $h_{q}^{v}$ is the representation of spot *q* in *H^v^*. Because the discrete assignments are non-differentiable, they are treated as fixed during backpropagation, whereas the ball centers remain differentiable with respect to their constituent representations.

Cross-View Association. Because granular balls are constructed independently in each view, their indices are not directly aligned. We therefore establish cross-view associations according to membership overlap. For granular balls $GB_{i}^{m}$ and $GB_{j}^{n}$, the normalized membership overlap and binary association are defined in Equation (10)

(10) $$\rho_{ij}^{\left(m,n\right)}=\frac{\left|I_{i}^{m}\cap I_{j}^{n}\right|}{\text{min}\;(n_{i}^{m},n_{j}^{n})},\;p_{ij}^{\left(m,n\right)}=I(\rho_{ij}^{\left(m,n\right)}>\tau_{o}),$$

where *τ_o_* is the overlap threshold, and $P^{\left(m,n\right)}=[p_{ij}^{\left(m,n\right)}]\in {\left\{0,1\right\}}^{B\times B}$ is the cross-view association matrix.

The indicator I(·) equals 1 when its argument is true and 0 otherwise. Since $\rho_{ij}^{\left(m,n\right)}=\rho_{ji}^{\left(n,m\right)}$, the association matrices satisfy the relation in Equation (11)

(11) $$P^{\left(n,m\right)}={\left(P^{\left(m,n\right)}\right)}^{⊤}.$$

Granular-Ball Contrastive Loss. For each unordered view pair (*m,n*), the stacked ball centers and positive-pair mask are defined in Equation (12):

(12) $$C^{\left(m,n\right)}=[\begin{array}{c} C^{m}\\ C^{n} \end{array}],\;M^{\left(m,n\right)}=[\begin{array}{cc} I_{B} & P^{\left(m,n\right)}\\ {\left(P^{\left(m,n\right)}\right)}^{⊤} & I_{B} \end{array}],$$

where $C^{v}={\left[c_{1}^{v},\ldots ,c_{B}^{v}\right]}^{⊤}$ and **I***_B_* denotes the *B* × *B* identity matrix. Following the implementation, the identity blocks designate within-view self-pairs as positives, whereas the off-diagonal blocks encode the cross-view associations identified by membership overlap.

Let ${\bar{c}}_{q}$ denote the *q*-th row of $C^{\left(m,n\right)}$. The temperature-scaled cosine similarity is defined in Equation (13)

(13) $$s_{qr}=\frac{{\bar{c}}_{q}^{⊤}{\bar{c}}_{r}}{\tau_{c}\|{\bar{c}}_{q}{\|}_{2}\|{\bar{c}}_{r}{\|}_{2}},$$

where *τ_c_ >* 0 is the temperature parameter. Following the mask-guided formulation used in the implementation, the loss for each view pair is defined in Equation (14)

(14) $$\begin{array}{l} {ℒ}^{\left(m,n\right)}=\frac{1}{\|M^{\left(m,n\right)}{\|}_{1}}\sum_{q=1}^{2B}\sum_{r=1}^{2B}M_{qr}^{\left(m,n\right)}\{\text{ log }[\text{exp }(s_{qr})\\ \;+\sum_{t=1}^{2B}\text{exp }\left(\left[1-M_{qt}^{\left(m,n\right)}\right]s_{qt}\right)]-s_{qr}\}. \end{array}$$

where $\|M^{\left(m,n\right)}{\|}_{1}$ denotes the sum of all entries in the binary mask. This objective promotes agreement between granular balls with sufficiently overlapping memberships while distinguishing them from unrelated structural units.

The overall granular-ball contrastive loss is given in Equation (15):

(15) $$L_{\text{GBCL}}=\frac{2}{V(V-1)}\sum_{1\leq m<n\leq V}L^{\left(m,n\right)}$$

### 3.4 Physical consistency refinement

The PhCR module preserves tissue topology in the fused representation *Z*. Here, physical consistency refers to the neighborhood relationships induced by the spatial coordinates of the spots. Let *z_i_* denote the latent representation of spot *i*. The cosine similarity between spots *i* and *j* in the latent-space cosine similarity is defined in Equation (16):

(16) $$H_{ij}=\frac{z_{i}^{⊤}z_{j}}{\|z_{i}{\|}_{2}\|z_{j}{\|}_{2}}.$$

Let $R_{i}^{+}$ and $R_{i}^{-}$ denote the sampled physical neighbors and non-neighbors of spot *i*, respectively. The PhCR loss is defined in Equation (17):

(17) $$L_{\text{R}}=-\sum_{i=1}^{N}[\sum_{j\in R_{i}^{+}}\text{log}\sigma (H_{ij})+\sum_{k\in R_{i}^{-}}\text{log}(1-\sigma (H_{ik}))],$$

where *σ*(·) is the sigmoid function. This objective encourages physically adjacent spots to exhibit similar latent representations while separating spots that are not physically connected. Consequently, the learned embedding preserves the local tissue organization encoded by the original spatial coordinates.

### 3.5 Overall loss function

MGCF-Net is trained end-to-end without a generative decoder. Its objective combines the MVRR, PhCR, and GBCL losses. The overall training objective is given in Equation (18):

(18) $$L=\lambda_{1}L_{\text{C}}+\lambda_{2}L_{\text{R}}+\lambda_{3}L_{\text{GBCL}},$$

where *λ*_1_, *λ*_2_, and *λ*_3_ are nonnegative hyperparameters that control the relative contributions of the three loss terms. The complete training procedure of MGCF-Net is summarized in **Algorithm 1**.

Algorithm 1

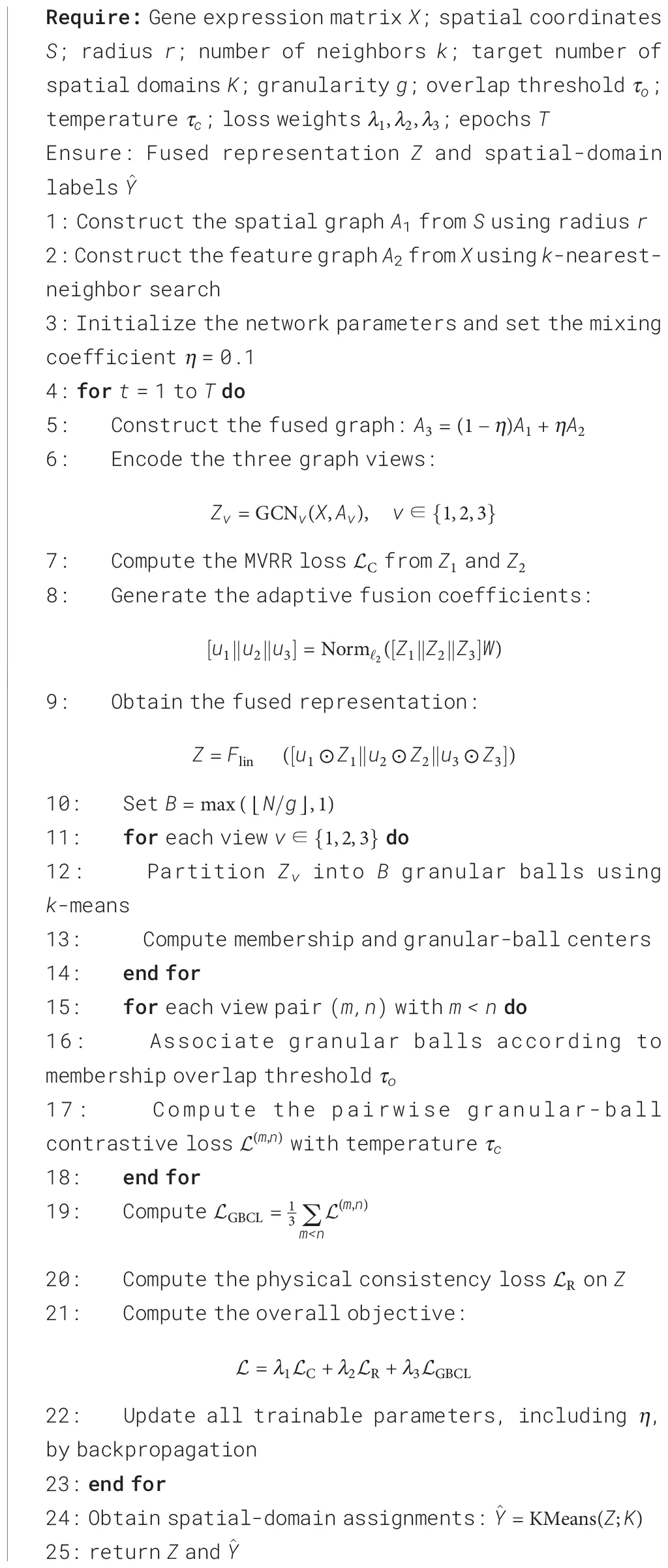

## 4 Experiments

We evaluate MGCF-Net in terms of clustering performance, spatial-domain visualization, component ablation, parameter sensitivity, and computational efficiency. All experiments use the human dorsolateral prefrontal cortex (DLPFC) dataset.

### 4.1 Experimental setup

#### 4.1.1 Dataset

We evaluate MGCF-Net on the human DLPFC spatial transcriptomics dataset generated using the 10x Genomics Visium platform Maynard et al. (2021). The dataset provides spatially resolved gene expression profiles and manual annotations of the cortical layers and white matter. For each tissue section, the target number of spatial domains *K* is fixed before training to the number of distinct manual annotation labels in that section. Only this count, rather than the spot-level annotation assignments, is supplied to MGCF-Net; the assignments are retained as reference labels for ARI and NMI evaluation. Our experiments include eight tissue sections: 151507, 151508, 151669, 151670, 151671, 151672, 151673, and 151674.

#### 4.1.2 Comparison methods

We compare MGCF-Net with eight representative spatial domain identification methods:

- *Scanpy* Wolf et al. (2018) is a conventional single-cell analysis framework that constructs a neighborhood graph from gene expression profiles and performs clustering through community detection.
- *SpaGCN* Hu et al. (2021) employs graph convolutional networks to integrate gene expression and spatial information for spatial domain identification.
- *stLearn* Pham et al. (2023) integrates gene expression and spatial information and can additionally incorporate histological image features to learn spatially informed representations.
- *DeepST* Xu et al. (2022) learns low-dimensional spot representations by jointly modeling spatial and transcriptomic information.
- *GraphST* Long et al. (2023) employs graph-based self-supervised learning to capture spatial dependencies and transcriptomic similarities.
- *SCGDL* Liu et al. (2023) identifies spatial domains through graph-based deep representation learning.
- *Spatial-MGCN* Wang et al. (2023) constructs multiple graphs to characterize spatial and transcriptomic structures.
- *stMMR* Zhang et al. (2024) integrates multiple information sources to learn spatial representations for domain identification.

For a fair comparison, all methods are evaluated using the same preprocessed expression matrix and spatial coordinates whenever supported by their implementations. We use the official implementations and recommended hyperparameter settings whenever available.

#### 4.1.3 Evaluation metrics

We use the adjusted Rand index (ARI) and normalized mutual information (NMI) to quantify the agreement between the identified spatial domains and the manual annotations Hubert and Arabie (1985); Strehl and Ghosh (2002). ARI measures pairwise assignment agreement while correcting for chance. An ARI value of 1 indicates perfect agreement, a value near 0 indicates chance-level agreement, and a negative value indicates agreement below chance. NMI quantifies the information shared between two partitions and ranges from 0 to 1. Higher ARI and NMI values indicate better clustering performance. In both tables, boldface and underlining mark the best and second-best results, respectively, with red and blue providing additional visual cues.

#### 4.1.4 Implementation details

To facilitate reproducibility, we report the computational environment used in our experiments. All experiments involving MGCF-Net were conducted on a workstation running Ubuntu. The workstation was equipped with a 24-core, 32-thread Intel Core i9-14900KF processor, 64 GB of DDR5 memory, a 2 TB NVMe M.2 solid-state drive, and two NVIDIA GeForce RTX 3090 GPUs with 24 GB of memory each.

### 4.2 Clustering performance

**Table 1** compares MGCF-Net with eight competing methods on eight DLPFC tissue sections in terms of ARI and NMI. MGCF-Net achieves the highest mean ARI of 0.61, outperforming stMMR, Spatial-MGCN, and GraphST, which achieve mean ARI values of 0.56, 0.55, and 0.53, respectively. Compared with stMMR, the best-performing baseline in terms of mean ARI, MGCF-Net yields an absolute improvement of 0.05 and a relative improvement of approximately 8.9%, based on the reported rounded means.

**Table 1:** Performance comparison of MGCF-Net and competing spatial transcriptomics methods on the DLPFC dataset.

Dataset	DeepST	GraphST	Scanpy	SCGDL	SpaGCN	Spatial-MGCN	stMMR	stLearn	MGCF-Net
ARI									
151507	0.55	0.48	0.20	0.49	0.39	**0.63**	0.59	0.49	**0.63**
151508	0.42	0.49	0.15	0.34	0.33	0.46	0.51	0.31	**0.56**
151669	0.44	0.48	0.10	0.24	0.23	0.39	0.49	0.32	**0.59**
151670	0.33	0.46	0.09	0.26	0.33	0.35	**0.48**	0.23	0.43
151671	0.52	0.61	0.12	0.31	0.42	0.60	0.68	0.39	**0.81**
151672	0.48	0.63	0.12	0.34	0.52	0.77	0.63	0.34	**0.80**
151673	0.54	**0.63**	0.20	0.33	0.40	0.61	0.60	0.30	0.61
151674	0.55	0.43	0.22	0.27	0.31	**0.60**	0.51	0.38	0.44
NMI									
151507	0.62	0.64	0.21	0.55	0.49	**0.74**	0.72	0.64	0.67
151508	0.57	0.54	0.21	0.44	0.43	0.60	0.65	0.53	**0.67**
151669	0.57	**0.59**	0.16	0.38	0.36	0.58	0.56	0.49	0.58
151670	0.51	**0.68**	0.16	0.36	0.43	0.56	0.56	0.41	0.54
151671	0.59	0.72	0.24	0.41	0.53	0.72	0.72	0.54	**0.74**
151672	0.60	0.61	0.23	0.46	0.60	0.75	0.72	0.47	**0.76**
151673	0.69	**0.74**	0.29	0.42	0.55	0.68	0.68	0.49	0.68
151674	**0.69**	0.61	0.31	0.38	0.46	**0.69**	0.62	0.54	0.58

Boldface and underlining mark the best and second-best results, respectively, with red and blue providing additional visual cues.

MGCF-Net achieves or ties for the highest ARI on five tissue sections: 151507, 151508, 151669, 151671, and 151672. On section 151507, MGCF-Net and Spatial-MGCN tie for the highest ARI, both achieving 0.63. **Figure 2** compares the spatial-domain assignments of MGCF-Net and the baseline methods with the manual annotation for section 151507. The largest improvements occur on sections 151669 and 151671, where MGCF-Net obtains ARI values of 0.59 and 0.81, exceeding the best baseline results by 0.10 and 0.13, respectively. It also increases the ARI from the best baseline value of 0.51 to 0.56 on section 151508 and from 0.77 to 0.80 on section 151672. The magnitude of the gain therefore varies across tissue sections.

**Figure 2:**
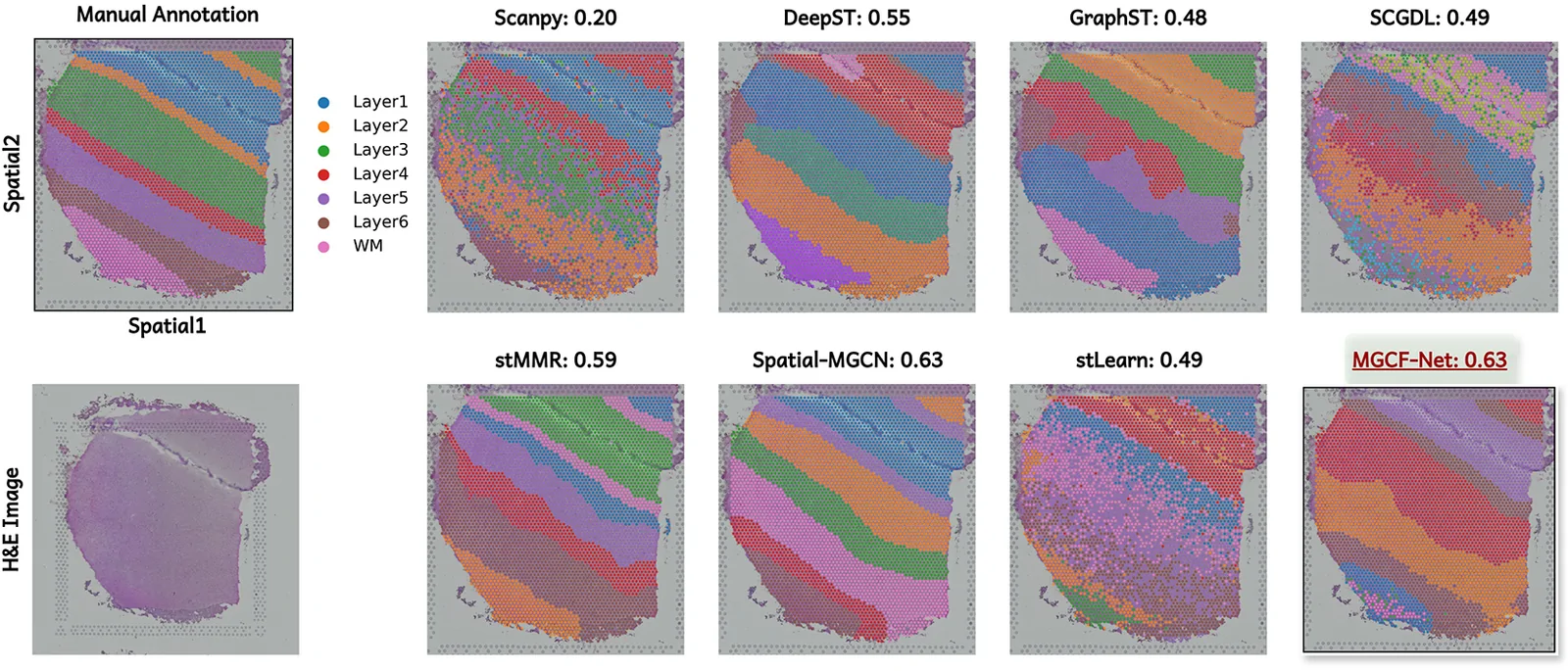
Spatial-domain identification results for MGCF-Net and the baseline methods on DLPFC section 151507. The numbers following the method names are ARI scores computed against the manual annotation. The H&E-stained image is included only as a morphological reference and is not used as input to MGCF-Net.

For NMI, MGCF-Net obtains a mean score of 0.65, compared with 0.65 for stMMR and 0.67 for Spatial-MGCN. It ranks first on sections 151508, 151671, and 151672, with scores of 0.67, 0.74, and 0.76, respectively, but does not lead on sections 151670 and 151674. Thus, MGCF-Net provides the highest mean ARI in this comparison while remaining competitive rather than uniformly superior in NMI.

### 4.3 Ablation study

To evaluate the contribution of each core component of MGCF-Net, we conduct ablation experiments on eight DLPFC tissue sections. We construct three variants, each with one component removed: (1) *w/o MVRR*, without multi-view redundancy reduction; (2) *w/o GBCL*, without granular-ball contrastive learning; and (3) *w/o PhCR*, without physical consistency refinement. All variants use identical network configurations and clustering protocols. The quantitative results are summarized in **Table 2**.

**Table 2:** Ablation study of the proposed model on eight DLPFC tissue sections.

Model Variant	ARI ↑	NMI ↑
w/o GBCL	0.5884	0.6519
w/o PhCR	0.5669	0.6371
w/o MVRR	0.5681	0.6507
**Full Model**	**0.609**	**0.653**

Boldface and underlining mark the best and second-best results, respectively, with red and blue providing additional visual cues.

The full MGCF-Net outperforms all ablated variants in both ARI and NMI. Disabling PhCR (*w/o PhCR*) produces the largest decreases in ARI (Δ = −0.0421) and NMI (Δ = −0.0159), which supports the use of explicit spatial constraints when assigning adjacent spots to coherent clusters. Removing MVRR reduces ARI and NMI by 0.0409 and 0.0023, respectively, consistent with its role in suppressing redundant cross-view correlations. Removing granular-ball structural supervision (*w/o GBCL*) also lowers both metrics, by 0.0206 in ARI and 0.0011 in NMI, linking group-level contrastive learning to the preservation of local topology.

The ablation pattern assigns distinct roles to the three components: MVRR supports representation discrimination, GBCL promotes local structural coherence, and PhCR reinforces physical consistency.

### 4.4 Parameter sensitivity analysis

We evaluate the sensitivity of MGCF-Net to the loss weights *λ*_1_, *λ*_2_, and *λ*_3_, which weight the MVRR, PhCR, and GBCL terms, respectively. Each parameter is selected from {10^−3^,10^−1^.^5^,10^0^,10^1^.^5^,10^3^}. We vary *λ*_3_ separately and jointly vary *λ*_1_ and *λ*_2_ across the DLPFC tissue sections. **Figures 3a, c** show moderate variation in mean ARI and NMI as *λ*_3_ changes. The error bars overlap substantially, so the variation associated with *λ*_3_ is smaller than the variation among tissue sections. A relatively small *λ*_3_ gives the highest mean ARI, but several values spanning multiple orders of magnitude produce similar scores.

**Figure 3:**
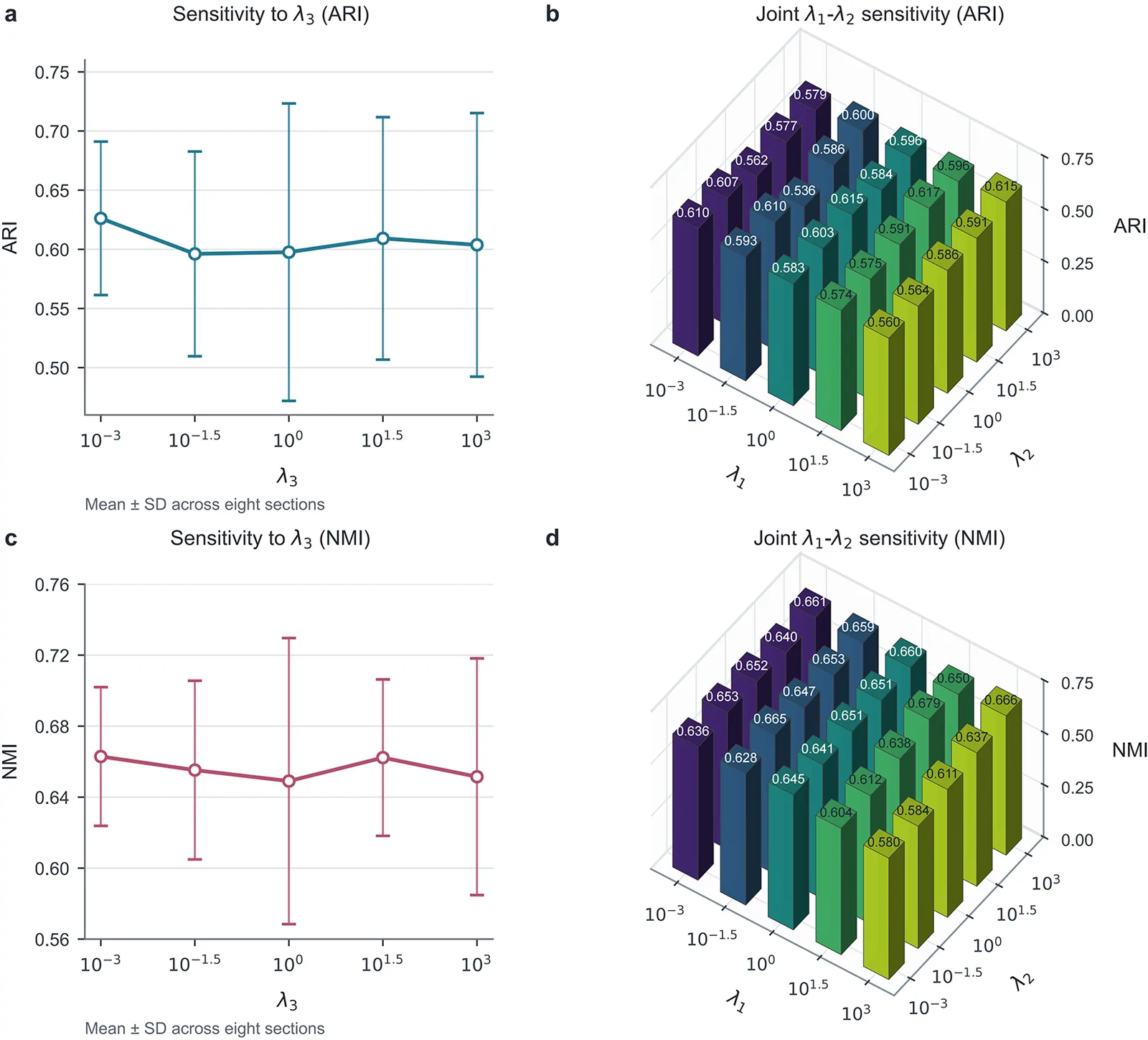
Parameter sensitivity of MGCF-Net on eight DLPFC tissue sections. **(a, c)** Effects of *λ*_3_ on ARI and NMI; points and error bars show the mean and standard deviation across the eight sections. **(b, d)** Joint effects of *λ*_1_ and *λ*_2_; bar heights show the mean across the same sections.

**Figures 3b, d** show no sharp degradation or isolated optimum across the tested combinations of *λ*_1_ and *λ*_2_. Within this grid, the balance between MVRR and PhCR can therefore change without a large shift in either metric. The tested loss weights form a broad plateau rather than a narrow optimum.

### 4.5 Running time analysis

We further compare the computational efficiency of MGCF-Net with that of representative spatial-domain identification methods on the DLPFC dataset. **Figure 4** reports the mean end-to-end runtime of the complete pipeline for each tissue section, with error bars representing the standard deviation across sections. Because the runtimes vary substantially among methods, the horizontal axis is shown on a logarithmic scale.

**Figure 4:**
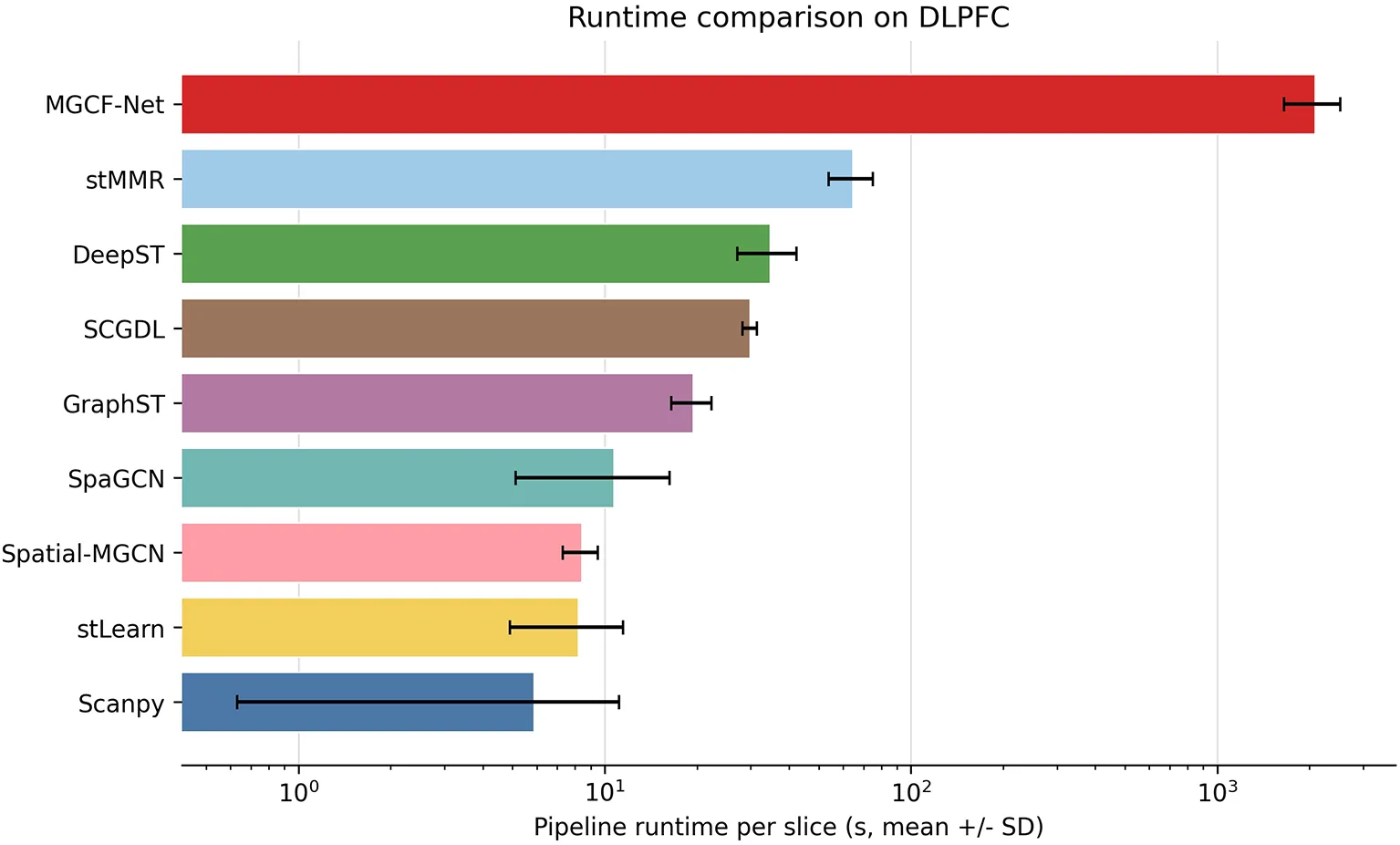
Comparison of the computational efficiency of MGCF-Net and representative spatial-domain identification methods on the DLPFC dataset. The runtime is measured as the mean end-to-end pipeline runtime per tissue section, and the error bars represent the standard deviation across sections.

**Figure 4** shows shorter runtimes for Scanpy, stLearn, Spatial-MGCN, and SpaGCN, whereas GraphST, SCGDL, DeepST, and stMMR incur additional computational overhead. MGCF-Net is the slowest evaluated method, requiring approximately 1×10^3^ seconds per tissue section on average. Its multi-stage representation learning procedure therefore carries a clear computational cost.

GBCL repeatedly partitions the representations from different views into granular balls and establishes cross-view associations based on membership overlap. Granular-ball construction, center computation, and contrastive optimization across multiple view pairs further increase the training cost. Future work may improve scalability by adopting sparse cross-view similarity computation, reducing the frequency of granular-ball updates, and employing more efficient sampling strategies.

## 5 Conclusion

This work presented MGCF-Net, a decoder-free multi-view graph learning framework for spatial domain identification in spatial transcriptomics. MGCF-Net integrates a spatial graph and a feature graph through view-specific encoding, MVRR, and spot-adaptive fusion. GBCL performs cross-view alignment over local structural groups, thereby reducing false-negative pairings and preserving coherent tissue structure. PhCR further regularizes spatial neighborhood relationships in the latent space.

Across eight DLPFC tissue sections, MGCF-Net obtains the highest mean ARI and competitive NMI among the evaluated methods. The ablation and sensitivity analyses characterize the contribution of each component and the response to the tested loss weights. Group-level structural contrastive learning thus offers an alternative to instance-level objectives and generative decoding for spatial representation learning. Future work will examine differentiable granular-ball construction, more scalable optimization, and extensions to three-dimensional and spatial multi-omics data.

## Data Availability

Publicly available datasets were analyzed in this study. This data can be found here: https://spatial.libd.org/spatialLIBD/.
